# Load-velocity relationship in the free-weight horizontal and incline bench press

**DOI:** 10.1038/s41598-025-12166-5

**Published:** 2025-07-19

**Authors:** Diogo Luís Marques, Waleed Abohasel, Shaea Alkahtani, Mohammed Alsaeed, Norah K. Algarzae, Henrique Pereira Neiva, Daniel Almeida Marinho, Mário Cardoso Marques

**Affiliations:** 1 Department of Sport Sciences, University of Beira Interior, Covilhã, 6200-001 Portugal; 2 Research Center in Sports Sciences, Health Sciences and Human Development, CIDESD, Covilhã, 6200-001 Portugal; 3 Department of Exercise Physiology, College of Sport Sciences and Physical Activity, King Saud University, Riyadh, 11451 Saudi Arabia; 4 Department of Biomechanics & Motor Behavior, College of Sport Sciences and Physical Activity, King Saud University, Riyadh, 11451 Saudi Arabia; 5 Department of Physiology, College of Medicine, King Saud University, Riyadh, Saudi Arabia

**Keywords:** Upper body, Strength, Movement velocity, Resistance training, Occupational health, Quality of life

## Abstract

This study compared (i) the load-velocity relationship in the free-weight horizontal (HBP) vs. incline bench press (IBP) and (ii) the differences between general vs. individual load-velocity equations to estimate the one-repetition maximum (1RM) in the HBP and IBP. Thirty males (26 ± 3 years) performed four sessions: two dedicated to assessing the 1RM in the HBP and IBP, and another two to measure the mean propulsive velocity (MPV) reached against loads of 40–90% 1RM in the HBP and IBP. Individual load-velocity equations estimated the MPV from 30 to 100% 1RM in the HBP and IBP and the 1RM in each exercise. Furthermore, general equations estimated the 1RM in each exercise. The estimated MPV values associated with 30–100% 1RM in the HBP and IBP were compared. The coefficient of variation (CV) and intraclass correlation coefficient (ICC) determined the reliability between the actual and estimated 1RM in both exercises. The results showed higher MPV in the HBP than in the IBP from 30 to 80% 1RM (*p* < 0.001). General and individual equations provided acceptable estimates of the 1RM when using loads from 70 to 90% 1RM in the HBP and IBP (CV < 10% and ICC > 0.80) but not 40–60% 1RM (CV > 10% and ICC < 0.80). This study shows that the load-velocity relationship differs between HBP and IBP, with higher velocities reached in HBP. Furthermore, individual and general equations seem reliable in estimating the 1RM when using relative loads from 70 to 90% 1RM in the HBP and IBP.

## Introduction

Over the last two decades, the measurement of movement velocity during resistance training has gained popularity in research and sports settings due to its accuracy in estimating the training load and individually monitoring the training effects^1,2^. Some of the first research studies showed a strong relationship (*r*^*2*^ ≥ 0.90) between the relative load (i.e., % of one-repetition maximum [1RM]) and movement velocity in the bench press^3–5^ and squat exercises^5,6^ performed in a Smith machine. The advantage of the latter equipment is that it stabilizes the execution technique and guarantees a linear movement of the barbell and the transducer’s cable, which can reduce error and increase the accuracy of data collection^7,8^.

On the other hand, in contrast to free-weights, one could argue that exercising with a Smith machine might decrease ecological validity^7,9,10^ as it limits the range of motion, reduces the engagement of synergistic muscles, and confines movement to a single plane of motion^11,12^. Nevertheless, it is important to note that recent research has demonstrated similar improvements in muscle strength and mass after resistance training using free-weights or a Smith machine^11,13,14^ thereby validating the use of both training modalities in sports contexts. The decision between exercising with free-weights or opting for the Smith machine ultimately rests on personal preferences and the specific adaptations an individual aims to promote based on their training goals^11,13,14^. From this perspective, exercising on a Smith machine may be suitable for controlling execution technique^15^ while free-weights might be effective in augmenting the involvement of synergistic muscles and positively impacting intermuscular coordination, thereby improving sports performance^11,13,15^.

Previous studies on the load-velocity profile in free-weight exercises have shown an acceptable degree of accuracy in the squat^15,16^ bench press^15,17–19^ deadlift^7^ and prone bench pull^20^. Indeed, Loturco et al.^18^ observed a similar accuracy level between the load-velocity profile in the Smith machine bench press (*r*^2^ = 0.97) and the free-weight bench press (*r*^2^ = 0.96), which supports the use of movement velocity to monitor resistance training load during free-weight exercises. When analyzing the differences in load-velocity profiles for bench press variations, including horizontal and inclined positions, evidence on this topic is scarce, with research primarily conducted on the Smith machine^19^. In this topic, previous evidence has shown higher lifting velocities in the inclined Smith machine bench press (45 degrees) compared to the horizontal position (average differences between exercises of 0.05 m·s^− 1^ in trained males), particularly pronounced with light to moderate relative loads^19^. Despite that, higher 1RM values in the horizontal Smith machine bench press were observed. These results suggest that a 45-degree inclination may increase the ability to displace low to moderate relative loads with higher velocity outputs, possibly due to the greater activation of the upper portion of the pectoralis major, anterior deltoid, and trapezius^19,21–23^. In comparison, the ability to displace high relative loads may be favored without bench inclination, possibly due to the higher activation of the triceps brachii, posterior deltoid, and the lower portion of the pectoralis major^19,21^. Nevertheless, considering that differences in the load-velocity profiles between bench press variations were observed for the Smith machine, where the movement is linear and more controlled, it would be important to understand whether the same results are consistent when using free-weights, taking into account the possible movement oscillations.

Another aspect to consider when modeling the load-velocity relationship is the choice between general or individual load-velocity regression equations. For instance, several studies have observed more accurate 1RM load estimates when modeling individual load-velocity regression equations in the free-weight bench press, squat, deadlift, and prone bench pull exercises^7,15,20,24^. Therefore, these results collectively suggest that individual regression equations might be chosen to model the load-velocity relationship in different resistance exercises, as they provide better adjustments between the relative load and movement velocity.

Given the abovementioned considerations, the current study analyzed the differences between the load-velocity relationship in the horizontal vs. incline free-weight bench press and the differences between general vs. individual load-velocity equations to estimate the 1RM. Based on previous literature analyzing the load-velocity profiles in the Smith machine bench press performed in the horizontal and inclined positions in trained males^19^ we hypothesized that the velocities reached against a broad spectrum of relative loads would be higher in the incline bench press than in the horizontal position. Furthermore, individual equations would produce more accurate estimates of the 1RM free-weight bench press load than the general equations, as observed in previous research^15^.

## Methods

### Study design

In a cross-sectional study of three weeks, the participants visited a laboratory six times, with two sessions per week. The first week was to measure body weight, height (CARDINAL DETECTO 339, USA), body fat, and fat-free mass (Inbody770, Biospace Co., Korea), and familiarize the participants with the exercises and testing procedures. In the second week, we conducted a direct assessment of the 1RM load in the free-weight horizontal and incline bench press exercises in separate sessions for each. Finally, in the third week, we measured the barbell velocity reached against loads ranging from 40 to 90% 1RM in the free-weight horizontal and incline bench press to develop general and individual load-velocity regression equations. All tests were performed in a counterbalanced order during the afternoon, separated by 72 h of rest. Two researchers supervised all testing procedures.

### Participants

Thirty Saudi males (26.4 ± 3.0 years, 72.6 ± 8.4 kg, 1.75 ± 0.07 m, 21.4 ± 4.0% body fat, 54.1 ± 7.6 kg fat-free mass) volunteered to participate in this study. The inclusion criteria consisted of physically active adult men who had previously performed the free-weight bench press exercise. The exclusion criteria were individuals with musculoskeletal injuries in the preceding three months. The participants self-reported that they were active and regularly played several sports, including football (21 participants), swimming (3 participants), and table tennis (6 participants). Nevertheless, none of the participants had previous experience in regular resistance training routines. All participants were advised to avoid strenuous efforts 24 h before each visit to the laboratory and consume the last meal 4 h before each test. Furthermore, all participants signed an informed consent to be part of the current research. The study protocol was approved by the Internal Review Board at King Saud University (IRB: E-21-6518) and follows the recommendations of the Declaration of Helsinki.

### Direct assessment of the one-repetition maximum in the free-weight horizontal and incline bench press

The direct assessment of the 1RM load followed the procedures described elsewhere^25^. After a general warm-up (i.e., 5 min of walking on a treadmill at a self-selected intensity, followed by upper-body mobility exercises), the participants performed a specific warm-up consisting of ten repetitions with a 10 kg load. Then, the test began with a weight of 20 kg, and the weight was progressively increased by 10 kg until the participants could perform only one maximal repetition within five attempts with an inter-set rest of 3 min. If the participant could not perform a single repetition, the load was decreased by 1–5 kg until he could achieve the 1RM load. The horizontal bench press was performed at an inclination of 0° and the incline bench press at 45° on a weight bench (Duojiu, Model Dj-0228, Fitness World Co., Jiangsu, China). The angles were confirmed through a goniometer (model 01135, Lafayette Instrument Co., USA). The participants performed the bench press while lying on the bench with their feet on the ground, grasping the bar with a pronated grip at shoulder-width apart. The barbell was lowered in a controlled manner (2–3 s) until it touched the chest. After touching, the concentric phase was performed at the maximal intended velocity (elbows fully extended). Two spotters, one on each side, ensured supervision and safety during the execution of the lifts.

### Indirect assessment of the one-repetition maximum through the measurement of movement velocity against six different relative loads in the free-weight horizontal and incline bench press

After performing the same general and specific warm-ups described previously for the direct assessment of the 1RM, participants performed one set of three repetitions with the maximal intended velocity against loads of 40%, 50%, 60%, 70%, 80%, and 90% of their 1RM load, determined in the previous week. This procedure enabled us to develop both general and individual load-velocity regression equations. We utilized 180 observations (30 participants multiplied by 6 relative loads and their associated velocities) to model the general load-velocity equations, and 6 observations (corresponding to the 6 relative loads and their associated velocities) to model the individual load-velocity equations. The barbell lifting technique and supervision remained the same as in the previous sessions, and the inter-set rest was 1 min. A linear velocity transducer (T-Force System, Ergotech, Spain) attached to the barbell measured the mean propulsive velocity of each repetition performed. This velocity variable reflects solely the propulsive phase of the movement (i.e., when the bar’s acceleration surpasses gravity’s acceleration), offering a more precise representation of neuromuscular capabilities compared to the conventional mean velocity (which also takes into account the braking phase), especially with low relative loads^26^. The fastest mean propulsive velocity attained against each relative load was kept for analysis^18^.

### Statistical analysis

The data normality was checked using the Shapiro-Wilk test, and descriptive statistics (means, standard deviations, and 95% confidence intervals) were calculated. A linear regression model was used to analyze the relationship between mean propulsive velocity and relative load in the horizontal and incline bench press. For the general equations, we used all observations from each participant (i.e., all velocities achieved at 40–90% 1RM) to create the linear model. For the individual equations, only the velocities achieved at 40–90% 1RM for each subject were included to create the regression model. The goodness of fit of the equations was assessed using the coefficient of determination (*r*^2^) and the standard error of the estimate (SEE). The individual regression equations allowed the estimation of the mean propulsive velocities associated with 30–100% 1RM in increments of 5% for both exercises. A cross-validation method was used on the general regression equations to assess the risk of overfitting. The dataset was split into two halves using the holdout approach, resulting in training and testing sets. Paired-samples t-test compared (i) the differences between the mean propulsive velocity values associated with each relative load in both exercises, (ii) the differences between the actual and estimated 1RM values with 40–90% 1RM in the horizontal and incline bench press, and (iii) the 1RM values estimated by the general and individual equations. The alpha level was set at *p* < 0.05. The intraclass correlation coefficient (ICC_3,1_) was used to analyze the relative reliability of the actual and estimated 1RM, while the coefficient of variation (CV) was used to assess the absolute reliability. ICC values were interpreted as poor (< 0.50), moderate (0.50–0.75), good (0.75–0.90), and excellent (> 0.90)^27^, while CV values < 10% were considered acceptable^28^. The SPSS v28 (SPSS Inc., United States) and Microsoft Excel^®^ (Microsoft Inc., Redmond, WA, United States) analyzed the data, and GraphPad Prism v7 (GraphPad Inc., San Diego, USA) plotted the data.

## Results

### Relationship between mean propulsive velocity and relative load in the free-weight horizontal and incline bench press

**Fig. 1:**
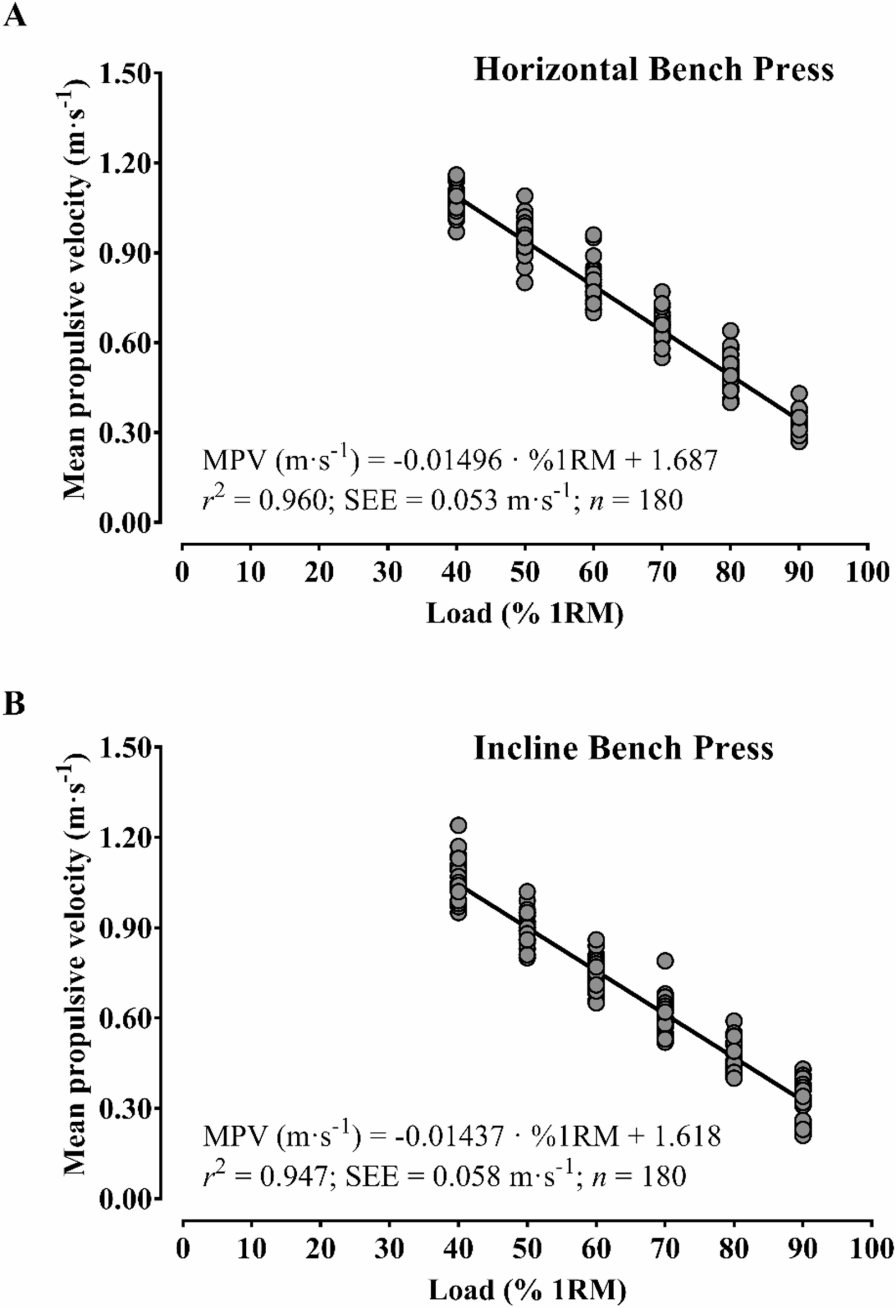
Load-velocity relationship in the horizontal (**A**) and incline bench press (**B**). MPV, mean propulsive velocity; RM, repetition maximum; SEE, standard error of the estimate.

Figure 1 shows very strong linear relationships between mean propulsive velocity and relative load (%1RM) in the horizontal (*r*^*2*^ = 0.96) and incline free-weight bench press (*r*^*2*^ = 0.95) exercises. The individual load-velocity relationships were also very strong in the horizontal (*r*^2^ values ranging from 0.95 to 0.99) and incline free-weight bench press (*r*^2^ values ranging from 0.94 to 0.99).

### Mean propulsive velocity values associated with each relative load

Table 1 presents the mean propulsive velocity values associated with relative loads ranging from 30 to 100% 1RM. The results showed higher mean propulsive velocities from 30 to 80% 1RM in the horizontal than in the incline bench press (*p* < 0.05).

**Table 1:** Differences between horizontal vs. incline bench press in the mean propulsive velocity values associated with each relative load.

Load	Horizontal Bench Press		Incline Bench Press		Differences	*p*-value^#^
% 1RM	Mean ± SD (m·s^− 1^)	95% CI (m·s^− 1^)	Mean ± SD (m·s^− 1^)	95% CI (m·s^− 1^)	Mean ± SD (m·s^− 1^)	
30	1.24 ± 0.06	1.22 to 1.26	1.19 ± 0.08	1.16 to 1.21	0.05 ± 0.08	< 0.001
35	1.16 ± 0.06	1.14 to 1.18	1.12 ± 0.07	1.09 to 1.14	0.05 ± 0.07	< 0.001
40	1.09 ± 0.05	1.07 to 1.1	1.04 ± 0.06	1.02 to 1.07	0.05 ± 0.06	< 0.001
45	1.01 ± 0.04	1.00 to 1.03	0.97 ± 0.05	0.95 to 0.99	0.04 ± 0.06	< 0.001
50	0.94 ± 0.04	0.93 to 0.95	0.90 ± 0.05	0.88 to 0.92	0.04 ± 0.05	< 0.001
55	0.86 ± 0.03	0.85 to 0.88	0.83 ± 0.04	0.81 to 0.84	0.04 ± 0.04	< 0.001
60	0.79 ± 0.03	0.78 to 0.80	0.76 ± 0.04	0.74 to 0.77	0.03 ± 0.04	< 0.001
65	0.71 ± 0.03	0.70 to 0.73	0.68 ± 0.03	0.67 to 0.70	0.03 ± 0.04	< 0.001
70	0.64 ± 0.03	0.63 to 0.65	0.61 ± 0.03	0.60 to 0.62	0.03 ± 0.04	< 0.001
75	0.57 ± 0.03	0.55 to 0.58	0.54 ± 0.04	0.53 to 0.55	0.02 ± 0.04	0.004
80	0.49 ± 0.03	0.48 to 0.50	0.47 ± 0.04	0.46 to 0.48	0.02 ± 0.05	0.022
85	0.42 ± 0.04	0.40 to 0.43	0.40 ± 0.04	0.38 to 0.41	0.02 ± 0.05	0.075
90	0.34 ± 0.04	0.33 to 0.36	0.33 ± 0.05	0.31 to 0.34	0.02 ± 0.06	0.184
95	0.27 ± 0.05	0.25 to 0.28	0.25 ± 0.06	0.23 to 0.27	0.01 ± 0.07	0.340
100	0.19 ± 0.05	0.17 to 0.21	0.18 ± 0.06	0.16 to 0.21	0.01 ± 0.08	0.518
All	0.79 ± 0.03	0.78 to 0.80	0.74 ± 0.03	0.74 to 0.77	0.03 ± 0.04	< 0.001

^#^ Paired-samples t-test; CI, confidence interval; RM, repetition maximum; SD: standard deviation.

### General load-velocity regression equations

The general equation for the horizontal bench press is the following: Load (%1RM) = 110.893 + (-64.187 · Mean Propulsive Velocity) (*r*^*2*^ = 0.96; SEE = 3.4% 1RM).

The proposed general equation for the incline bench press is the following: Load (%1RM) = 110.128 + (-65.965 · Mean Propulsive Velocity) (*r*^*2*^ = 0.95; SEE = 3.9% 1RM).

Table 2 presents the results of the cross-validation analysis for the general equations. The data indicate the absence of overfitting, as evidenced by the elevated correlation coefficient values (*r* = 0.973–0.985) and the negligible differences between the two subsets.

**Table 2:** Cross-validation using the holdout method.

	Relative load (% 1RM)	Testing set*	Training set*
Mean propulsive velocity	Horizontal bench press	0.974	0.985
	Incline bench press	0.973	0.973

* Pearson correlation coefficient between predicted and observed values.

### Actual vs. estimated one-repetition maximum using general and individual equations

There were differences between the actual and estimated 1RM through the general and individual equations when using the velocities associated with 40, 50, 60, 70, and 80% 1RM in the horizontal bench press (*p* < 0.001) and the velocities associated with 40, 50, 60, 70, 80%, and 90% 1RM in the incline bench press (*p* < 0.001). On the other hand, there were no differences in the estimated 1RM values through the general and individual equations across all relative loads in the horizontal and incline bench press (*p* > 0.05).

The results showed lower differences and higher reliability levels (i.e., higher ICC and lower CV values) between the actual and estimated 1RM using both general and individual equations when velocities were associated with loads of 70–90% 1RM (Table 3).

**Table 3:** Differences between the actual and estimated 1RM using general and individual equations.

	Horizontal Bench Press		Incline Bench Press	
	General EQ	Individual EQ	General EQ	Individual EQ
1RM_actual_ (kg)	53.9 ± 10.5	53.9 ± 10.5	52.5 ± 10.2	52.5 ± 10.2
1RM_est_-40% (kg)	62.6 ± 10.8	63.2 ± 9.5	65.4 ± 13.8	66.2 ± 11.8
1RM_actual_ – 1RM_est_ (kg)	-8.7 ± 10.0	-9.3 ± 8.2	-12.9 ± 10.0	-13.7 ± 8.9
ICC_(3,1)_	0.43 (-0.01-0.71)	0.47 (-0.07-0.76)	0.42 (-0.10-0.74)	0.38 (-0.11-0.72)
CV (%)	12.9 ± 8.9	13.3 ± 7.2	15.5 ± 9.8	17.2 ± 8.7
1RM_est_-50% (kg)	62.5 ± 12.9	62.5 ± 12.0	60.4 ± 11.4	60.6 ± 10.4
1RM_actual_ – 1RM_est_ (kg)	-8.6 ± 7.4	-8.6 ± 5.4	-7.9 ± 6.0	-8.1 ± 5.2
ICC_(3,1)_	0.64 (-0.02-0.87)	0.69 (-0.07-0.90)	0.67 (-0.05-0.89)	0.67 (-0.08-0.89)
CV (%)	10.4 ± 8.0	10.6 ± 5.7	10.2 ± 6.6	10.7 ± 5.6
1RM_est_-60% (kg)	59.5 ± 13.2	59.2 ± 12.1	58.4 ± 12.0	58.4 ± 11.8
1RM_actual_ – 1RM_est_ (kg)	-5.6 ± 6.6	-5.3 ± 5.1	-5.9 ± 4.8	-5.9 ± 4.6
ICC_(3,1)_	0.77 (0.30–0.91)	0.81 (0.21–0.94)	0.80 (0.06–0.94)	0.80 (0.04–0.94)
CV (%)	7.3 ± 6.3	10.6 ± 5.7	7.4 ± 5.7	7.4 ± 5.3
1RM_est_-70% (kg)	58.0 ± 11.0	57.8 ± 10.3	57.6 ± 10.9	57.4 ± 10.8
1RM_actual_ – 1RM_est_ (kg)	-4.1 ± 3.6	-3.9 ± 3.1	-5.1 ± 4.5	-4.9 ± 3.6
ICC_(3,1)_	0.88 (0.29–0.96)	0.89 (0.22–0.97)	0.82 (0.14–0.94)	0.85 (0.08–0.96)
CV (%)	5.6 ± 3.8	5.3 ± 3.9	6.9 ± 5.5	6.6 ± 4.5
1RM-80% (kg)	56.7 ± 12.3	56.4 ± 11.6	56.8 ± 11.0	56.5 ± 11.1
1RM_actual_ – 1RM_est_ (kg)	-2.8 ± 3.4	-2.5 ± 2.8	-4.3 ± 3.4	-4.0 ± 2.9
ICC_(3,1)_	0.93 (0.68–0.98)	0.95 (0.71–0.98)	0.88 (0.18–0.96)	0.90 (0.18–0.97)
CV (%)	4.0 ± 3.3	3.7 ± 2.6	6.0 ± 3.6	5.5 ± 3.1
1RM_est_-90% (kg)	55.1 ± 10.9	54.6 ± 10.4	55.6 ± 10.5	55.2 ± 10.7
1RM_actual_ – 1RM_est_ (kg)	-1.2 ± 1.8	-0.7 ± 2.1	-3.1 ± 3.4	-2.7 ± 2.7
ICC_(3,1)_	0.98 (0.94–0.99)	0.98 (0.95–0.99)	0.90 (0.55–0.97)	0.94 (0.60–0.98)
CV (%)	2.1 ± 1.7	2.2 ± 1.7	4.9 ± 3.4	3.9 ± 2.8

Data are presented as mean ± standard deviation. 1RM_actual_ – 1RM_est_ indicates the differences between the actual and estimated 1RM. The intraclass correlation coefficient model ICC_(3,1)_ is presented with 95% confidence intervals; CV, coefficient of variation; RM, repetition maximum.

## Discussion

This study aimed to compare the load-velocity profiles in the horizontal vs. incline free-weight bench press and to examine the differences between general and individual load-velocity equations in estimating the 1RM load. The main findings of this study indicated that (i) higher mean propulsive velocity values were reached in the horizontal than in the incline free-weight bench press at relative loads from 30 to 80% 1RM, and (ii) general and individual equations provided acceptable estimates of the 1RM load when using relative loads from 70 to 90% 1RM but not from 40 to 60% 1RM in recreationally active Saudi males without regular participation in resistance training.

Contrary to our first study hypothesis, the results indicated higher movement velocities in the free-weight horizontal bench press compared to the inclined position (average differences of 0.03 m·s^− 1^). These results differ from prior research^19^ which indicated that trained males achieved higher movement velocities in the inclined position than in the horizontal position, with average differences of approximately 0.05 m·s^− 1^ between exercises. However, it should be noted that participants in that study performed both bench press variants in a Smith machine, which may be a significant factor biasing the comparison to our results. When comparing the load-velocity profiles in both bench press variants derived in the study by García-Ramos et al.^19^ with the results of the present study, one can notice average differences of 0.02 m·s^− 1^ in the horizontal position and 0.07 m·s^− 1^ in the inclined position. Considering the similarity of the load-velocity profiles in the horizontal bench press performed on the Smith machine and with free-weights, it can be speculated that the equipment may not significantly interfere with this relationship, as long as the remaining conditions are kept relatively identical, such as execution technique, use of the barbell, as well as the age range and sex of participants. The level of resistance training experience may not be a differentiating factor in this relationship, considering that previous evidence with top-level athletes^18^ demonstrated a very similar load-velocity profile in the free-weight bench press compared to our results with individuals who do not regularly participate in resistance training. In that study, the reported average mean propulsive velocities associated with ~ 45%, ~ 55%, ~ 65%, ~ 75%, ~ 85%, and ~ 95% 1RM were 1.01 m·s^− 1^, 0.87 m·s^− 1^, 0.72 m·s^− 1^, 0.58 m·s^− 1^, 0.38 m·s^− 1^, and 0.26 m·s^− 1^, respectively^18^. These velocities represent an average difference of ~ 0.01 m·s^− 1^ compared to our results. Although these are average values, which do not account for the individual profiles of each athlete, they are still relevant to this discussion due to their proximity. Interestingly, the study by Loturco et al.^18^ found similar velocities associated with nearly all relative loads in the free-weight and Smith machine bench press variants, except for those corresponding to ~ 45% and 55% 1RM. In these cases, the velocities reached in the Smith machine (1.08 m·s^− 1^ with 45% 1RM and 0.92 m·s^− 1^ with 55% 1RM) outperformed those reached with free-weights (1.01 m·s^− 1^ with 45% 1RM and 0.87 m·s^− 1^ with 55% 1RM). These findings suggest that performing the same strength exercise on the Smith machine results in higher velocity outputs with lower relative loads. This occurrence is likely due to the enhanced stability and technical control provided by the machine.

On the other hand, it can be speculated that the complexity or higher level of technical difficulty involved in performing the free-weight incline bench press, compared to the Smith machine variant and horizontal position, may have limited individuals’ ability to express their full potential when executing repetitions at maximal intended velocity, particularly with low and moderate relative loads. These hypothetical limitations may be more pronounced in individuals who do not regularly participate in resistance training. Associated with the prior observation is the fact that the horizontal oscillations that may occur during free-weight exercises, which can be more pronounced during the incline bench press due to its technical demands, can negatively affect measurements made with the linear transducer^29,30^. The horizontal oscillations in the barbell can lead to inaccuracies in calculated velocities and the respective estimations of the load-velocity profiles^29,30^. Therefore, considering the discrepancies in velocities achieved in the incline bench press between the present study and previous data, future research is needed to analyze the pattern of load-velocity profiles in horizontal and incline bench press exercises performed using a Smith machine and free-weights in young adult men.

This study found that the best estimation of the 1RM load was at a higher relative 1RM. Some methods of predicting 1RM bench press are accurate, and the strength of this relationship can be influenced by the relative loads assessed (i.e., 30 to 95% of 1RM)^4^. The results of our study suggest that higher relative loads or slower mean propulsive velocities yield the most reliable 1RM estimates in the horizontal and incline free-weight bench press in recreationally active males. Therefore, the velocities associated with 70–90% 1RM should be chosen to estimate the 1RM when using the general equations proposed in our study.

Surprisingly, our results showed no significant differences between the accuracy of general and individual load-velocity equations in estimating the 1RM load for both the horizontal and incline free-weight bench press exercises. This finding was unexpected, given that previous studies have reported more accurate 1RM estimates when using individualized equations^7,15,20,24^. One possible explanation for these results is that our participants presented similar 1RM strength levels and comparable resistance training experience, which may have minimized variability and led to similar outcomes regardless of the equation used. Given this context, it is plausible that the difference between general and individual load-velocity equations may be negligible for individuals with limited training experience. Consequently, using general equations could serve as a practical and efficient alternative in such populations. It is important to note, however, that although the regression equations used in this study were developed in Saudi Arabia, they may still be applicable in other regions of the Arab Gulf. Beyond these areas, their applicability may be limited, and further research is needed to validate their use across diverse populations. Moreover, the general use of load-velocity regression equations must be approached with caution. Significant estimation errors may arise if the context and purpose for which the equations were initially developed are not considered. The load-velocity relationship is influenced by several individual factors, including but not limited to sex, age, type of strength exercise, and equipment used, which must be accounted for to ensure accuracy^31^. Therefore, for more precise 1RM estimation and effective training monitoring, it is advisable to develop individualized load-velocity regression equations that incorporate these key variables and reflect the unique characteristics of each athlete.

This study has limitations that must be addressed. First, although the participants reported being physically active, none of the participants reported regular participation in resistance training, and their low relative strength reflects a lack of muscle strength development. Therefore, providing an extended familiarization period would have been important to improve their general strength and execution technique, thereby increasing the feasibility of the experimental procedures, especially in a technically more demanding exercise like the incline bench press. Second, although we have provided a 72-hour rest between 1RM testing sessions, the 1RM procedure may be problematic in some situations because after failing an attempt to reach the 1RM load, subjects accumulate excessive fatigue, which can contribute to underestimating their actual 1RM. Furthermore, considering the frequent changes in the 1RM load^1^it is possible that the weights deviated slightly from the programmed relative loads in the third week. In this respect, measuring the velocity during the 1RM testing session would have been important to establish their load-velocity profiles and guarantee that in the third session, the velocities reached against each weight matched the programmed relative loads (40–90% 1RM). Additionally, one could have used the velocity associated with the 1RM load to model the individual load-velocity profiles, as this calculation was only performed with velocities associated with relative loads of 40–90% 1RM, which may have resulted in some inaccuracies in predicting the velocities associated with 95% and 100% 1RM. Finally, the absence of a comparison of load-velocity profiles in both the incline and horizontal bench press, utilizing the Smith machine and free-weights, precludes drawing more robust assertions regarding potential differences and similarities in execution techniques and exercise variants. Consequently, this limitation keeps the topic open for further exploration.

## Conclusions

This study demonstrated that recreationally active males achieved higher velocities in the free-weight horizontal bench press than in the inclined position at relative loads ranging from 30 to 80% 1RM. Furthermore, no differences were found between general and individual regression equations for estimating the 1RM load in horizontal and incline free-weight bench press exercises at relative loads of 70–90% 1RM. Therefore, since no differences were observed between individual and general load-velocity equations in both bench press positions, the current equations can be applied to predict the 1RM load among Saudi adults without a resistance training background. Applying such equations can help strength and conditioning coaches in Saudi Arabia empirically track their athletes’ strength progress in every single session without the need for performing a demanding and time-consuming 1RM test. As a final note, it is essential to consider that the use of these general regression equations may be less precise when testing different inexperienced subjects, especially those from diverse regions of the globe. In contrast, the accuracy of individual relationships in estimating relative loads and monitoring training intensity should remain unaffected.

## Data Availability

All the data is available in the manuscript.
